# Platelets, extracellular vesicles and coagulation in pulmonary arterial hypertension

**DOI:** 10.1177/20458940211021036

**Published:** 2021-06-04

**Authors:** Sarah Cullivan, Claire A. Murphy, Luisa Weiss, Shane P. Comer, Barry Kevane, Brian McCullagh, Patricia B. Maguire, Fionnuala Ní Ainle, Sean P. Gaine

**Affiliations:** 1 National Pulmonary Hypertension Unit, Mater Misericordiae University Hospital, Dublin, Ireland; 2 Conway-SPHERE Research Group, Conway Institute, University College Dublin, Dublin, Ireland; 3 Department of Neonatology, Rotunda Hospital, Dublin, Ireland; 4 Department of Haematology, Mater Misericordiae University Hospital, Dublin, Ireland

**Keywords:** pulmonary circulation, pulmonary hypertension, platelets, extracellular vesicles, coagulation, thrombosis

## Abstract

Pulmonary arterial hypertension is a rare disease of the pulmonary vasculature, characterised pathologically by proliferation, remodelling and thrombosis *in situ*. Unfortunately, existing therapeutic interventions do not reverse these findings and the disease continues to result in significant morbidity and premature mortality. A number of haematological derangements have been described in pulmonary arterial hypertension which may provide insights into the pathobiology of the disease and opportunities to explore new therapeutic pathways. These include quantitative and qualitative platelet abnormalities, such as thrombocytopaenia, increased mean platelet volume and altered platelet bioenergetics. Furthermore, a hypercoagulable state and aberrant negative regulatory pathways can be observed, which could contribute to thrombosis in situ in distal pulmonary arteries and arterioles. Finally, there is increasing interest in the role of extracellular vesicle autocrine and paracrine signalling in pulmonary arterial hypertension, and their potential utility as biomarkers and novel therapeutic targets. This review focuses on the potential role of platelets, extracellular vesicles and coagulation pathways in the pathobiology of pulmonary arterial hypertension. We highlight important unanswered clinical questions and the implications of these observations for future research and pulmonary arterial hypertension-directed therapies.

## Introduction

The current clinical classification categorises pulmonary hypertension (PH) into five distinct groups, based on shared clinical characteristics, pathophysiology and predicted treatment response.^1^ Group 1 pulmonary arterial hypertension (PAH), the focus of this review, is characterised by progressive dyspnoea, fatigue and ultimately right heart failure if left untreated. The pathology of PAH is characterised by intimal and adventitial fibrosis, smooth muscle proliferation, plexiform lesions and thrombosis.^2,3^ Indeed, the frequent observation of thrombosis in situ led to considerable interest in the role of anticoagulation and antiplatelet agents as potential therapeutic targets. However, their routine empiric use is not currently recommended due to the overall low level of evidence.^4–6^ Nevertheless, evidence for abnormalities in platelets, extracellular vesicles (EVs) and coagulation have mounted in recent years. Anomalies in platelet count, size and function have been described in PAH. Furthermore, elevated levels of circulating signalling molecules that are usually stored in platelet granules such as platelet-derived growth factor (PDGF), transforming growth factor beta (TGF-β) and serotonin have frequently been observed.^7^ Increased EV levels from diverse cellular origins have also been noted and circulating levels have been correlated with endothelial dysfunction and haemodynamic parameters.^8,9^ Additional abnormalities in thrombin generation and reduced serum thrombomodulin have been observed.^10^ This review summarises available evidence regarding the role of platelets, EVs and coagulation in the pathobiology of PAH and the implications for future research and therapeutic interventions.

## Platelets in PAH

### Platelet count, size and function

Platelets are small, anucleate blood cells, with diverse roles in a number of physiological processes including haemostasis, thrombosis and immune function.^11^ Circulating platelets have a typical lifespan of 7–10 days and are much smaller than their megakaryocyte progenitors, with an average diameter of 2–5 μm and a mean platelet volume (MPV) between 6 and 10 fl.^12^ Abnormalities in platelet count, size and function have been described in PAH. Thrombocytopaenia is frequently observed in PAH, in both patients who are treatment naïve and those on prostacyclin therapy.^13–15^ In idiopathic PAH (IPAH), thrombocytopaenia is associated with worse haemodynamics^15^ and reduced survival in incident patients.^14,16^ Therefore, the platelet count may have a role in multiparametric risk assessment and prognostic stratification in IPAH.^14,16^ The underlying mechanism for this is uncertain; however, increased peripheral platelet destruction due to shear stress in remodelled pulmonary vessels may be responsible.^17^ While it has been suggested that platelet sequestration may occur in advanced right heart failure due to hepatic congestion and portal hypertension, platelet counts are not associated with typical indicators of right heart failure, such as right atrial pressure.^14^

Furthermore, MPV is increased in patients with IPAH when compared to healthy controls.^13,18^ This may have implications for platelet function, as larger platelets are considered more active. Activated platelets are involved in diverse biological processes, and can mediate thrombosis, immune responses and release mitogenic and vasoactive mediators.^17,19^ Relevant to the field of PAH is the role of platelets in the transcellular metabolism of eicosanoids and thromboxane production, as thromboxane is a potent vasoconstrictor and levels are typically elevated in PAH.^20^ Furthermore, platelets contain numerous intracellular granules that store molecules that have been implicated in PAH. Platelet alpha granules store a number of important molecules that are typically elevated in PAH, including platelet factor-4 (PF4), PDGF, vascular endothelium growth factor (VEGF), TGF-β, p-selectin and von Willebrand factor (vWF).^21,22^ Serotonin is stored in platelet-dense granules and is also implicated in the pathobiology of PAH.

Serotonin came to the attention of the PH community in the 1960s due to an epidemic of anorexigen-associated PAH (APAH),^17^ and elevated levels were subsequently observed in other PAH subgroups.^23^ Serotonin is metabolised in the liver and lungs and transported via the serotonin transporter (SERT), which is present in various cell types including pulmonary artery smooth muscle cells (PASMCs) and platelets. Under normal circumstances, circulating levels of serotonin are low, as excess could promote harmful inflammation, proliferation and vasoconstriction in the pulmonary circulation.^24^ Disruption of serotonin homeostasis by selective serotonin receptor uptake inhibitors was associated with increased clinical worsening events and mortality in PAH.^25^ Furthermore, increased SERT activity has been observed in PASMC.^26^ Interestingly, defective platelet serotonin storage can persist despite ‘curative’ heart–lung transplantation.^27^

Finally, there is considerable interest in metabolic dysfunction in PAH and the potential role of circulating platelets as a surrogate to explore this. Platelets from patients with PAH have a distinct metabolic phenotype when compared to healthy controls. This is defined by increased basal glycolysis and reduced glycolytic reserve.^28,29^ Interestingly, platelet bioenergetics has been correlated with haemodynamic parameters, including mean pulmonary artery pressure and pulmonary vascular resistance (PVR).^28^ These quantitative and qualitative platelet abnormalities highlight the potential role of platelets in the pathobiology of PAH and the importance of further studies in this field.

### Megakaryocytes in PAH

Platelet production and size are regulated at the level of the megakaryocyte. While the bone marrow was historically considered the primary site of thrombopoiesis, there is increasing evidence that thrombopoiesis may occur in additional sites, including the pulmonary microcirculation.^30,31^ Megakaryocytes may have difficulty passing through pulmonary capillaries due to their large size, with an average diameter of 7–10 μm, and may become wedged at pulmonary vascular bifurcations. Intravascular pulmonary megakaryocytes are frequently observed in lung pathology specimens^32^ and local thrombopoiesis has been suggested by studies demonstrating higher levels of megakaryocytes in precapillary blood and higher platelet counts in postcapillary blood.^30,31,33^ Nevertheless, the subject of pulmonary thrombopoiesis remains controversial and requires further clarification.^33–35^ Furthermore, elevated levels of thrombopoietin, a glycoprotein hormone that regulates thrombopoiesis, have been demonstrated in a study of 14 patients with PAH, with the highest levels measured in the pulmonary arteries during right heart catheterisation.^36^ In vitro evidence suggests that individual platelet release from megakaryocyte cytoplasmic extensions termed proplatelets may be enhanced by intravascular shear stress.^33,37^ While the role of pulmonary megakaryocytes in PAH remains unclear, their involvement is plausible, as they could contribute to local stasis, thrombosis, EV secretion and inflammatory cytokine release.^38–40^

### EVs in PAH

EVs were first identified as ‘platelet dust’ in 1967 by Peter Wolf and since then our understanding of these diverse and complex particles has continued to evolve.^7,41^ EVs are small, anucleate, membrane-bound vesicles that can be released from numerous cell types, including platelets, megakaryocytes, erythrocytes, endothelial and inflammatory cells.^7,41^ EVs are important mediators of cell-to-cell communication. Their properties depend on the characteristics of the parent cell, their origin within that cell and stimuli in the local environment at the time of formation.^42^ EVs have numerous biological functions in health and disease, including effects on vascular tone, coagulation, inflammation and angiogenesis, which they mediate via cell surface proteins and through the release of soluble mediators.^42,43^ Platelet EVs, for example, can express tissue factor and phosphatidylserine on their surface membranes, resulting in procoagulant properties that are considerably more potent than their parent platelet. Furthermore, platelet EVs can release a variety of soluble mediators that are typically stored in platelet granules including PF4, vWF, VEGF and serotonin.^7^ A variety of definitions have been employed to describe EVs. If the subcellular origin is clear, terms such as exosomes (EVs of endosomal origin) and ectosomes (cell membrane derived EVs termed microparticles or microvesicles) can be used. Where there is any ambiguity regarding their origin, then EVs should be described based on physical and biological characteristics, to facilitate subsequent interstudy comparisons.^41^ There is evolving evidence for the role of EV autocrine and paracrine signalling in PAH, and their potential utility as biomarkers and novel therapeutic targets.

Human PASMCs and pulmonary vascular endothelial cells are intimately implicated in the pathobiology of PAH. EV-mediated communication and micro RNA transfer between these cell types has been demonstrated in vitro.^44,45^ Using novel methodology, de la Cuesta et al. have demonstrated the uptake and translation of PASMC-derived EVs by human pulmonary endothelial cells, providing biological evidence for EV-mediated crosstalk between these important cell types. Furthermore, human PASMC-EVs contain TGF-β superfamily ligands, such as growth differentiation factor 11 (GDF11) and TGF-β3, which are implicated in the pro-fibrotic and pro-proliferative phenotype observed in PAH. TGF-β signalling can also alter human PASMC-EV cargo and enhance EV-mediated micro RNA transfer.^45^ These findings suggest a potential paracrine effect of EVs on TGF-β signalling and provide exciting opportunities to explore the pathological and therapeutic consequences of this pioneering work.^45^

EVs may also serve as novel biomarkers in PAH and increased circulating levels have been correlated with endothelial dysfunction^46^ and haemodynamic parameters.^8,9^ In 20 patients with IPAH, significantly higher levels of EVs expressing tissue factor and endothelial (CD105+) markers were noted, with the highest levels in patients with more severe disease.^8^ This was defined as a six-minute walk distance (6MWD) < 380 m and NYHA functional class (FC) III or IV.^8^ Increased T cell (CD3+)-derived EVs were demonstrated in patients with IPAH and hereditary PAH (HPAH) when compared to controls.^47^ Increased platelet-derived EVs (CD42a, CD42b) were shown in patients with IPAH, which were higher in male patients. These subsequently decreased with epoprostenol therapy.^48^ In a study of 80 patients with PAH, platelet and leukocyte EV concentration were significantly lower in individuals prescribed prostacyclin analogues when compared to those who were not.^49^ Interestingly, this effect differed between prostacyclin agents, as epoprostenol was associated with decreased EV concentrations, while treprostinil had no significant effect on EV levels.^49^

The role of circulating EVs in the pathobiology of PAH is plausible. Indeed, they are important regulators of endothelial function, inflammation and coagulation, and levels can fluctuate with disease severity and therapeutic interventions in patients with PAH.^42^ Further research is required to clarify the role of EVs in risk stratification models and treatment algorithms; however, recent efforts to standardise EV definitions should facilitate future collaborate research efforts.^41^

### Coagulation in PAH

The pathology of PAH is characterised by intimal and adventitial fibrosis, smooth muscle proliferation, plexiform lesions and thrombosis in situ.^2^ Thrombotic lesions are frequently observed in distal muscular pulmonary arteries in PAH, though the precise origin and significance of these remains somewhat controversial.^17^ Thrombosis in situ may be an epiphenomenon, reflecting a local response to multiple stimuli, including shear stress, endothelial dysfunction, pro-inflammatory and pro-coagulant signals and aberrant negative regulatory pathways. It is conceivable that this thrombosis subsequently drives disease progression and contributes to increased PVR. Irrespective, a number of abnormalities in coagulation have been described in individuals with PAH that could promote thrombosis.^2^ These include altered tissue factor expression, vWF levels and increased thrombin generation.

Thrombin generation is primarily initiated by the extrinsic coagulation pathway, when exposed tissue factor binds and activates factor VII and triggers downstream cascades. Thrombin cleaves fibrinogen to fibrin during clot formation, to release fibrinopeptide A. These reactions occur on the surface of platelets and endothelium. Healthy endothelium typically does not express tissue factor, as increased tissue factor expression could result in excess thrombin generation and harmful intravascular coagulation. Interestingly, tissues factor expression is influenced by numerous factors, including inflammation, shear stress and hypoxia, all of which can be abnormal in PAH. Increased tissue factor antigen expression has been demonstrated in plexiform lesions from human subjects with PAH^50^ and tissue factor expressing EVs have been correlated with disease severity in IPAH.^8^ There is some evidence from preclinical studies that soluble guanylate cyclase (sGC) agonists inhibit the expression of tissue factor, which may account for some of the clinical benefits of these drugs.^51^

Abnormalities in vWF composition and levels have been described in PAH. vWF is synthesised by endothelial cells and is composed of low, intermediate and high-molecular-weight multimers. It acts as a carrier protein for factor VIII and has important roles in platelet adhesion and aggregation. Increased levels of dysfunctional vWF has been demonstrated in PAH, with loss of high-molecular-weight multimers.^52^ This was attenuated by exogenous prostacyclin therapy.^52^ Subsequent studies have shown low and low–normal levels of vWF antigen and activity in PAH.^53,54^ These results were attributed to an acquired von Willebrand syndrome in the context of a ‘high-shear high-flow’ circulation.

Abnormalities in thrombin activity have also been observed in PAH. Eisenberg et al. first demonstrated increased fibrinopeptide A levels in 31 patients with primary pulmonary hypertension (PPH) in 1990, indicating increased thrombin activity.^55^ Increased thrombin formation was subsequently demonstrated by Tournier et al. in 16 patients with IPAH, using a method called calibrated automated thrombography.^10^ Curiously, reduced thrombin generation was later shown using the same method by Vrigkou et al.^53^ These conflicting results may reflect differences in sample preparation (platelet rich plasma versus platelet poor plasma) and patient population (IPAH versus IPAH and connective tissue disease-associated PAH), and will require additional investigation. Indeed this could signal that increased thrombin generation is isolated to specific subgroups of PAH, which may account for differing responses to anticoagulation observed in some retrospective studies.^56^ Importantly, the role of thrombin extends beyond fibrin clot formation.^57^ Prolonged thrombin exposure is associated with alterations in nitric oxide–cyclic guanosine monophosphate signalling, endothelial cell migration and myofibroblast differentiation.^57,58^ Furthermore, thrombin cleaves protease-activated receptor-1, which is implicated in numerous processes that ultimately culminate in vasoconstriction.

In order to prevent inappropriate thrombin generation, a number of natural anticoagulants exist including tissue factor pathway inhibitor (TFPI), activated protein C (APC) and antithrombin.^59^ Alterations in the levels and activity of some of these factors have also been reported in patients with PAH. TFPI inhibits the activity of tissue factor through the formation of a TFPI/FXa/FVIIa/tissue factor complex and both increased^10^ and normal TFPI activity^60,61^ have been demonstrated. Another important pathway that prevents excess thrombin generation is the APC pathway. APC is formed when thrombomodulin on vascular endothelial cells binds and inactivates thrombin and forms a complex that catalyses the formation of APC. In the presence of the cofactor protein S, APC can then inactivate coagulation factors Va and VIIIa. Reduced levels of soluble thrombomodulin^10,62^ have been consistently demonstrated in patients with PAH, with levels increasing with parenteral prostacyclin therapy.^62^ Soluble thrombomodulin is formed when endothelial-bound thrombomodulin is cleaved in the presence of cytokines and neutrophils. Interestingly soluble thrombomodulin retains some of its cofactor activity and therefore lower levels may be relevant in PAH and suggest reduced anticoagulant activity. The fibrinolytic pathway has also been implicated in PAH, with evidence of increased plasminogen activator inhibitor-1.^63,64^

A review of coagulation in the pulmonary vasculature would be incomplete without reference to heparin. The lung is a rich source of heparin due to an abundance of mast cells. Heparin exerts anticoagulant activity indirectly via antithrombin III and subsequent inhibition of factors Xa and IIa.^65^ It also has antiinflammatory, antiproliferative and antiviral properties.^66^ Nebulised heparin has been studied in a range of respiratory conditions including asthma,^66^ acute lung injury^67^ and viral infections such as COVID-19.^68^ It can inhibit PASMC^69^ and pulmonary vascular pericyte^70^ proliferation and augment endothelial nitric oxide bioavailability.^71^ Presently, heparin does not have a defined role in the treatment algorithm of PAH.

## Anticoagulation, antiplatelets and novel therapeutics in PAH

### Anticoagulant therapy

The consistent observation of thrombosis in situ and organised, recanalised thrombi in the pathological specimens of patients with PPH^4,5,72^ led to immense interest in the therapeutic role of anticoagulation in this disease. A retrospective study of 120 patients with PPH published by Fuster et al. in 1984 demonstrated a significant survival benefit associated with anticoagulation therapy, which prompted further research in this area.^5^ The potential beneficial effects of anticoagulation in specific PAH subgroups appears intuitive, in light of objective evidence of thrombosis in pathological specimens, increased thrombin generation and augmented platelet activation. However, the current evidence supporting anticoagulation use is largely retrospective, observational and provides inconsistent and conflicting results. Therefore empiric anticoagulation in PAH subgroups such as IPAH, HPAH and APAH has somewhat fallen out of vogue and there is an urgent need for well-designed prospective controlled trials to address this uncertainty.

A systematic review of 12 non randomised studies published in 2018 suggested a potential survival benefit of anticoagulation in IPAH, but reduced survival in systemic sclerosis-associated PAH.^56^ This review included both the COMPERA registry data,^73^ which suggested a survival benefit with anticoagulation in IPAH, and the REVEAL cohort study,^74^ which failed to show a survival benefit in this subgroup. Current guidelines reflect this uncertainty. The 2015 European Society of Cardiology/European Respiratory society guidelines for the diagnosis and treatment of PH, advised consideration of oral anticoagulation with warfarin in patients with IPAH, HPAH and APAH (class IIb, level C).^1^ The 6th World Symposium on Pulmonary Hypertension in 2018 reiterated this recommendation, and emphasised that decision making should be individualised, and advised against anticoagulation in associated forms of PAH.^75^ The American College of Chest Physicians declined to make formal recommendations in their 2019 guidelines due to persistent ambiguity regarding the clinical benefits.^76^ Therefore in clinical practice, decisions regarding empiric anticoagulation for patients with IPAH, HPAP and APAH are made on a case by case basis, in expert centres. Anticoagulation is not recommended for other types of PAH unless there is an alternative indication.^75^ At present, there is no evidence for the use of direct oral anticoagulants in PAH.

### Antiplatelet therapy

Recognised imbalances in thromboxane and prostacyclin signalling in PAH, which are important regulators of platelet activation and aggregation, indicated a potential role for antiplatelet therapy in PAH. A randomised clinical trial of aspirin and simvastatin for PAH (ASA-STAT), including 65 patients, was terminated prematurely due to futility. This failed to demonstrate a significant effect on the primary end point of 6MWD at six months.^77^ In this study, there was a 93% reduction in thromboxane B2 levels, which is less than the anticipated 97–99% reduction that is typically observed with aspirin therapy. The authors suggest that this may reflect aspirin resistance or an aspirin-independent source of thromboxane production in individuals with PAH.^77^ Platelet inhibition with antiplatelet therapy has not translated to tangible clinical benefits and therefore antiplatelet therapy is not currently recommended.

### Additional therapies

PAH is characterised by an imbalance of vasoactive mediators, with an excess of thromboxane and endothelin (ET) and a relative deficiency of prostacyclin and nitric oxide. Restoration of this balance is the cornerstone of modern PAH therapy, with established therapies targeting the nitric oxide, prostacyclin pathways and ET pathways.

Nitric oxide and prostacyclin are important negative regulators of platelet activation, adhesion, and aggregation and reduced levels are consistently observed in PAH.^78^ It is biologically plausible that reduced bioavailability of nitric oxide and prostacyclin in the pulmonary circulation could lead to excess local platelet activation and drive disease progression.^11^ Evidence suggests that the phosphodiesterase type 5 inhibitor sildenafil^79^ and the sGC stimulator riociguat^78^ restore nitric oxide-mediated inhibition of platelet aggregation. Similarly, the beneficial effects of prostacyclin therapy may be partly mediated by antiplatelet effects and modulation of specific EV concentrations.^49,80^

Conversely, ET-1 activity is increased in PAH and mediates diverse effects including vasoconstriction, remodelling and proliferation via ET_A_ and ET_B_ receptors.^81^ While the direct role of ET-1 in coagulation is unclear, six months of dual ET receptor antagonism with macitentan in patients with PAH due to congenital heart disease was associated with improvements in coagulation abnormalities.^82^ Thromboxane levels are also elevated in PAH, which is relevant to this review as thromboxane is a potent vasoconstrictor, platelet agonist and mitogen. Therefore there is immense interest in thromboxane inhibition as an additional therapeutic target. Terbogrel is an oral thromboxane synthase inhibitor and thromboxane receptor antagonist that failed to improve 6MWD or haemodynamic parameters in subjects with PAH and NYHA FC II and III symptoms.^83^ However, interest in thromboxane inhibition persists and a novel thromboxane receptor antagonist NTP42 has demonstrated promising results in preclinical studies.^84^

There is considerable interest in the exploration of additional therapeutic pathways in PAH, including therapies that might attenuate platelet and coagulation abnormalities. Elevated circulating serotonin in individuals with IPAH and APAH prompted considerable interest in serotonin as a potential therapeutic target.^11,27^ However, studies targeting this pathway have so far have been disappointing, including a phase 2 study of the serotonin 2A/2B receptor antagonist terguride, which failed to show a clinical benefit in subjects with PAH. Further studies exploring the serotonin hypothesis, with a specific focus on tryptophan hydroxylase 1 are planned.^85^

Imatinib is a tyrosine kinase inhibitor which inhibits breakpoint cluster region-abelson (BCR-ABL) and additional kinases such as PDGF receptors α and β. Aberrant PDGF signalling is considered an important factor in the pathobiology of PAH and therefore it is plausible that tyrosine kinase inhibition may attenuate pulmonary vascular remodelling.^86^ The Imatinib in Pulmonary Arterial Hypertension, a Randomised, Efﬁcacy Study trial demonstrated improved exercise capacity and haemodynamics with imatinib therapy over a 24-week study period.^87^ However, serious adverse events were common and included subdural haematoma in patients who were prescribed concurrent anticoagulation. This may be mediated in part by reduced platelet aggregation.^88^

Sotatercept is a novel, first-in-class recombinant fusion protein that is undergoing evaluation in PAH.^89^ Sotatercept binds activins and GDFs to restore homeostasis between pro- and antiproliferative signals in the TGF-β superfamily pathway, which are imbalanced in PAH. In the PULSAR trial, treatment with sotatercept was associated with a significant reduction in PVR. Haematological adverse events were anticipated and noted, including thrombocytopaenia and polycythaemia.^89^ Indeed sotatercept has previously been studied as a potential treatment of anaemia in patients with haematological malignancy.^90^ The precise mechanism of thrombocytopaenia and associated clinical implications require exploration.

## Conclusion

PAH is a rare, incurable and progressive disease of the pulmonary vasculature, which is characterised by numerous haematological derangements, including thrombocytopaenia, increased MPV and altered platelet bioenergetics. Furthermore, increased thrombin generation and reduced negative regulatory pathways have also been described. Increased EVs from diverse cellular origins have been observed and include EVs with a potential paracrine effect on TGF-β signalling. These provide exciting opportunities to explore the pathological and therapeutic consequences of EVs in PAH subgroups. Due to the paucity of available evidence, widespread use of empiric aspirin or anticoagulation is not recommended, though there is some evidence that targeted PAH medications including parenteral epoprostenol, ET receptor antagonists and sGC stimulators may attenuate some of these derangements. High quality translational studies and clinical trials are required to further elucidate the role of platelets, coagulation and EVs in the pathobiology of PAH and their potential as biomarkers and therapeutic targets.

## Take home message

This review focuses on the potential role of platelets, extracellular vesicles and coagulation pathways in the pathobiology of PAH and highlights important unanswered clinical questions regarding the role of antiplatelet therapy and anticoagulation.
